# Inhalation-Based Nanoparticle Drug Delivery Targeting the Diseased Lower Airways in Idiopathic Pulmonary Fibrosis

**DOI:** 10.3390/pharmaceutics18020168

**Published:** 2026-01-27

**Authors:** Jin Woong Lee, Melissa Skibba, Tyler Tang, Hyeran Noh, Allan R. Brasier, Seungpyo Hong

**Affiliations:** 1 Pharmaceutical Sciences Division, University of Wisconsin, Madison, WI 53705, USA; jlee2266@wisc.edu (J.W.L.); ttang55@wisc.edu (T.T.); 2 Endocrinology Division, School of Medicine and Public Health, University of Wisconsin, Madison, WI 53705, USA; skibba2@wisc.edu; 3 Department of Optometry, Seoul National University of Science and Technology, Seoul 01811, Republic of Korea; hrnoh@seoultech.ac.kr; 4 Carbone Cancer Center, University of Wisconsin, Madison, WI 53705, USA; 5 Institute for Clinical and Translational Research, University of Wisconsin, Madison, WI 53705, USA; 6 Wisconsin Center for NanoBioSystems, University of Wisconsin, Madison, WI 53705, USA; 7 Lachman Institute for Pharmaceutical Development, University of Wisconsin, Madison, WI 53705, USA

**Keywords:** chronic airway diseases, idiopathic pulmonary fibrosis, pulmonary barriers, inhalable nanomedicine, therapeutic modalities, clinical translation

## Abstract

Initiated in the lower airways, idiopathic pulmonary fibrosis (IPF) is a fatal disease that disrupts the lung’s functional architecture, for which therapeutics are of limited efficacy; consequently, the disease is progressive and incurable. New therapeutic approaches providing delivery of mechanism-modifying drugs directly to the diseased regions may maximize therapeutic effects while minimizing systemic exposure. In this context, inhalable nanomedicine is an emerging approach for targeted pulmonary delivery, enabling a highly localized therapeutic effect. However, successful clinical translation is hindered by complex biological and engineering challenges in the diseased lungs, including region-specific clearance mechanisms, mucosal airway obstruction, microenvironmental remodeling, and disrupted aerodynamics of particle deposition. This review highlights these critical obstacles in the context of lower airway pathology, focusing on the growing understanding of the epithelial–mesenchymal transition, basal lamina remodeling, and fibroblastic heterogeneity in IPF. Therapeutic payloads, including small molecules, antibodies, and peptides, are compared in terms of stability, targeting, and tissue access. We further discuss emerging nanoparticle-based strategies designed to overcome these pulmonary barriers, with a focus on dendron micelles, dendrimer–peptide conjugates, lipopeptides, and biological vesicles. Finally, we explore advances in formulation engineering and aerosol generation technologies that are shaping the path toward clinically translatable inhalable nanomedicines.

## 1. Introduction

Chronic airway diseases, including idiopathic pulmonary fibrosis (IPF), chronic obstructive pulmonary disease (COPD) and allergic asthma (AA), collectively constitute a major global health challenge [1]. Although their etiologies differ, all of these chronic diseases initiate in lower airways and result in remodeling of the normal airway architecture. IPF is a non-resolving interstitial lung disease that is diagnosed in approximately 17.7 per 100,000 people in the world [2]. Separately, COPD currently ranks as the third leading cause of death globally, affecting over 200 million (M) people [3], and AA currently affects approximately 260 M people worldwide [4]. These estimates indicate that lower airway remodeling diseases produce a staggering, global impact in morbidity, mortality and health care expenditures.

IPF, COPD, and AA share a common pathogenic mechanism of tissue remodeling [5,6]. Tissue remodeling is a multicellular process initiated by airway epithelial injury from environmental oxidants, viral infections, and/or allergen-induced mucosal activation. These injuries produce epithelial damage, leading to disruption of the protective airway barrier function. This epithelial turnover leads to basal (progenitor) cell expansion to repopulate the barrier and restore homeostasis. In disease, repetitive injury produces persistence of dysplastic progenitor cells and exaggerated extracellular matrix (ECM) deposition, which disrupt airway mechanics, producing impaired gas exchange and reduced exercise tolerance [5,6]. Recent mechanistic work has shown that airway remodeling originates in the small airways of the lung (<2 mm diameter) [7]. Within the small airways, specialized sentinel bronchiolar cells play key roles in initiating and sustaining remodeling responses to inhaled toxicants and allergens [8]. In IPF, high-resolution imaging has shown that terminal dilation and epithelial hypertrophy are seen in small airways, preceding interstitial fibrosis [9].

This review focuses specifically on IPF, a disease characterized by a pattern of interstitial fibrosis produced by invasive, activated myofibroblasts arising from defined epithelial “niches” in the lower airway, resulting in irreversible scarring, disruption of the alveolar structure, and a subsequent loss of lung function [10,11]. Although pirfenidone, nintedanib and nerandomilast are currently the FDA-approved treatments for IPF, slow the progression or manage symptoms, they are not curative [12,13]. These treatments are also frequently associated with significant systemic side effects that reduce medication adherence, ultimately limiting therapeutic efficacy [14].

This existing therapeutic gap has motivated researchers, clinicians, and pharmaceutical companies to investigate novel delivery strategies. An attractive solution is to engineer strategies for delivering potent therapeutic agents directly to specific pathological sites within the small airways utilizing the vast epithelial surface of the lungs, which spans approximately 100 m^2^ in adults [15]. This inhalation therapy approach, in theory, can enhance therapeutic effects while reducing systemic exposure to non-affected tissues [15,16,17]. Consequently, advancing novel inhalable drug delivery systems to overcome these hurdles and improve patient outcomes has become a major area of research [16,18,19].

Several recent reviews have summarized idiopathic pulmonary fibrosis biology, and others have summarized inhaled nanocarrier platforms for pulmonary drug delivery [20,21,22,23]. However, these topics are often discussed separately, and the implications of small airway remodeling for aerosol deposition, clearance, epithelial access, and retention within fibrotic lesions are not always made explicit. To address this need, the present review connects current evidence on distal airway remodeling, including epithelial transitional states, basal cell expansion, ECM remodeling, and fibroblast heterogeneity, with practical formulation and device considerations that determine regional delivery and clinical translation of inhaled nanomedicines for idiopathic pulmonary fibrosis [19,24,25].

## 2. Major Challenges for Inhalable Nanomedicine Targeting Lower Airways

Effective regional delivery in IPF requires nanoparticle systems that can (i) overcome region-specific biological defenses; (ii) achieve predictable deposition in diseased small airways; and (iii) navigate the dynamic microenvironmental remodeling characteristic of IPF.

### 2.1. Region-Specific Clearance Mechanisms

The normally sterile airway is functionally divided into four anatomically distinct regions: oropharynx/trachea, bronchioles, and alveoli. Each region exhibits distinct mechanisms to trap and clear aerosolized pathogens or particles from penetrating into the interstitial fluid [26,27,28]. Large-diameter inhaled particles are filtered in the oropharynx and the bronchioles by being trapped by high molecular weight mucinous glycoproteins (mucus), facilitating their clearance by ciliary escalator activity through the coordinated ciliary action known as the mucociliary escalator [28,29]. Smaller particles entering the alveoli are bound by pulmonary surfactants, promoting their engulfment by sentinel alveolar macrophages [27]. Small molecules may also clear rapidly after deposition. They can dissolve in airway lining fluid and be absorbed across the air–blood barrier, lowering local exposure. This motivates formulations that prolong lung residence when sustained local action is needed [15,30]. In addition to these passive clearance mechanisms, inhaled nanoparticles must evade binding to intrinsic pattern recognition receptors whose engagement activates innate pulmonary defense resulting in undesired inflammation and airway remodeling [8]. The lung also contains drug-metabolizing enzymes and transporters. These can locally inactivate or efflux susceptible drugs, further reducing epithelial exposure even when deposition is achieved [31,32]. Aspects of these barriers relevant to nanoparticle delivery are detailed below.

Mucociliary clearance in the upper airway: Differentiated epithelium in the oropharynx and bronchioles secrete a complex viscoelastic mesh of high molecular weight mucinous glycoproteins, primarily mucin 5AC (MUC5AC) and mucin 5B (MUC5B) [33]. These mucin fibers create a physical sieve that sterically hinders the diffusion of nanoparticles into the underlying airway mucosa [34]. Beyond simple size exclusion, nanoparticles are often immobilized by mucin fibers through adhesive interactions, including hydrophobic and electrostatic forces [28]. Once trapped, these particles are efficiently and rapidly cleared by the mucociliary escalator described above [35]. This mechanism presents a formidable barrier that prevents nanoparticle penetration and subsequent therapeutic delivery to the upper airway [36].

**Figure 1:**
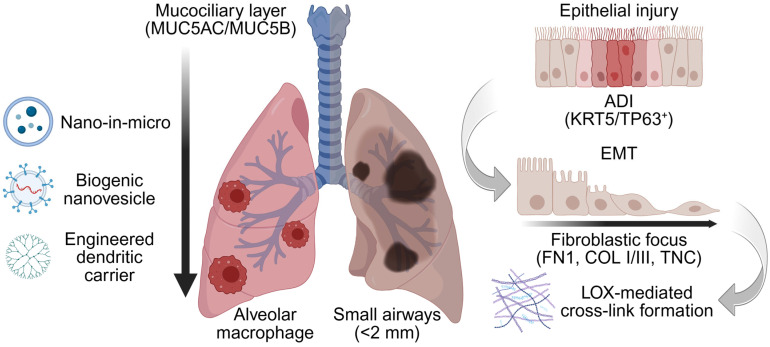
Barrier-aware design context for inhaled nanomedicine in the diseased lower airways. Representative inhalable nanocarrier classes considered in this review (nano-in micro, biogenic nanovesicles/exosomes, and dendritic nanocarriers). In the diseased lung, alveolar loss and microenvironmental changes including epithelial–mesenchymal transition (EMT) repopulate the airway with alveolar differentiation intermediates (ADI, KRT5/TP63^+^), accompanied by basal lamina and interstitial remodeling characterized by fibronectin 1 (FN1) scaffolding, type I/III collagen deposition, and lysyl oxidase (LOX)-mediated cross-linking. These features define airway-specific barriers (mucus, macrophage clearance, remodeled ECM) and motivate the development of barrier-aware surface chemistries. nano-in-micro aerodynamic packaging, and imaging-guided readouts developed in subsequent sections. The vertical arrow denotes the proximal-to-distal airway axis, highlighting the shift from mucociliary clearance in the conducting airways to macrophage-dominated clearance in the distal lung. Created in BioRender. Lee, J. (2026) https://BioRender.com/0lxxd1i (accessed on 22 January 2026).

Macrophage clearance in the lower airway: Lacking ciliary defenses, resident macrophages clear inhaled materials entering the lung distally [37]. Particles reaching the alveoli are coated by surfactants, C-reactive protein and apolipoproteins, abundant in the alveolar lining fluid [38]. This “corona” effect marks them for macrophage uptake and clearance. This rapid and efficient engulfment can eliminate over 90% of deposited particles, directly undermining the therapeutic goals of achieving a prolonged residence time in the lower airways [39]. Consequently, strategies to evade alveolar macrophage opsonization are essential for sustained alveolar action of any inhaled nanomedicine [40]. Although these biological defenses set the stage, deposition physics governs which airway generations are actually reached during breathing maneuvers. These airway-specific defenses, including the MUC5AC/MUC5B mucus mesh and alveolar macrophage uptake, are summarized in Figure 1.

### 2.2. Aerodynamic Properties Governing Deposition

The successful delivery of therapeutics to the lower airways is governed by aerosol physics, which are highly dependent on the particle’s mass median aerodynamic diameter (MMAD) [30]. The MMAD dictates how a particle behaves within the airstream as it navigates the respiratory tract’s branching structure. Particles with an MMAD greater than 5 µm possess high inertia and cannot follow branching bifurcations of the upper airways, leading to inertial impaction and particle deposition in the oropharynx and large bronchi [41]. Conversely, particles smaller than 1 µm have very low inertia and remain suspended in the airstream, reaching the alveoli. However, these particles are exhaled before deposition can occur via diffusion (Brownian motion) [42]. Therefore, an optimal MMAD between 1 and 5 µm is required [43]. In this range, particles are small enough to bypass impaction in the upper airways but large enough to deposit efficiently in the smaller bronchioles and alveoli through sedimentation under the force of gravity as airflow velocity decreases [30]. Achieving this specific size range is a critical engineering challenge for effective lower airway targeting [44]. The favorable 1–5 µm aerodynamic window and the relevance of targeting small airways (<2 mm) are shown in Figure 1. Beyond generic barriers, disease-driven remodeling redefines pore structure, stiffness, and cell states, requiring condition-specific design rules. Additionally, IPF can create poorly ventilated regions. These areas receive little deposition due to the airflow of aerosols, and delivery may bias toward better-ventilated tissue [45,46].

### 2.3. IPF Remodeling Barriers to Inhaled Nanomedicine

Beyond these common physiological barriers, IPF remodeling introduces additional constraints on aerosol deposition, clearance, and nanocarrier transport that must be considered in delivery approaches. IPF is a relentlessly progressive interstitial lung disease characterized by bronchiolization (lower airway), honeycombing (airway), and fibroblastic foci (FF) within a stiffened extracellular matrix [10]. These changes reduce compliance and disrupt oxygenation, reshaping transport barriers for inhaled therapeutics.

#### 2.3.1. IPF Is Initiated by Lower Airway Injury

Mechanistically, IPF represents an exaggerated wound-healing response to lower airway epithelial damage [10]. Factors triggering epithelial injury include infections from Epstein–Barr virus, herpesvirus, or exposures to cigarette smoke, silicate dust, and burn pit emissions [47,48]. These injuries induce lower airway epithelial cell death, senescence, and stem cell depletion [49,50,51]. Repopulation of the airway is from basal cells originating from specialized “niches” within the broncho-alveolar duct junction and alveolus. These progenitor populations initiate re-epithelialization by de-differentiating through a coordinated cellular reprogramming event known as EMT. EMT produces a loss of apical polarity and enables cytokinesis, allowing these progenitors to migrate and re-establish epithelium in injured distal airways [52].

Highly resolved, three-dimensional analyses of lung microarchitecture in IPF indicate that the earliest pathological changes originate in the small airways. Here, terminal bronchiolar branching is reduced, with thickened, phenotypically abnormal epithelium; importantly, all these changes precede the onset of microscopic fibrosis [53]. These mechanistic studies indicate that therapeutic targeting of the small bronchiolar epithelium is likely to disrupt the progression of the disease. These injury-repair processes make epithelial target availability and intracellular processing stage-dependent. Therefore, targeting and releasing strategies should be aligned with the evolving distal-airway epithelial state [52,53].

#### 2.3.2. Dysregulation of Mucociliary Clearance Mechanisms

The normal barrier functions of the airway detailed previously are significantly affected by IPF. In IPF, the normal branching of the small airways is disrupted, with bronchiolar drop-out and honeycomb cyst formation disrupting alveolar architecture and airflow [10]. At a microscopic level, epithelial injury-repair reduces the population of ciliated cells, disrupting ciliary motility, a process that reduces the ability of the lower airway to clear particulates [54]. In parallel, submucosal glands of the bronchi hypertrophy to secrete increased amounts of MUC5B and MUC5AC producing alveolar mucus plugging in diseased areas [55]. Understanding and overcoming these dynamic changes in mucociliary clearance and mucus composition will be important strategies for developing successful inhalational therapy in IPF. Collectively, altered airway architecture and mucus transport can shift residence time and create patchy deposition. Inhaled nanocarriers should be tested under disease-relevant clearance conditions rather than healthy-airway assumptions [10,54,55].

#### 2.3.3. Dynamic Changes in the Lower Airway Epithelium

In IPF, repetitive micro-injuries restructure the epithelium with a population of dystrophic ADI cells through a process of EMT. ADI cells outcompete the normal alveolar type II population and colonize the alveoli, disrupting gas exchange [56,57]. Spatial transcriptomics and lineage tracing studies have shown that these ADI cells are actively maintained by trophic Notch interactions with the activated ECM-secreting myofibroblast population [58,59]. These findings provide novel targets to modulate pathogenic cellular “niches” in this disease. As differentiation stalls, changes in the basal lamina alter both binding opportunities and transport resistance. The sequence from epithelial injury to EMT and formation of ADI is illustrated in Figure 1. This epithelial restructuring can change which cells take up inhaled nanocarriers, so targeting should be validated in IPF-relevant epithelial models [56,57,58,59].

#### 2.3.4. Dynamic Changes in the Basal Lamina Composition

Histologically, lower-airway remodeling in IPF shows patchy honeycombing and FF with deposition of a dense, stiff ECM. In the normal lower airway, the epithelial basal lamina is rich in laminins (LAM), fibronectin (FN), proteoglycans, and collagens (COL), providing structural support and differentiation cues. Although basal lamina has been considered relatively static, laser-capture microdissection studies reveal a distinct basal lamina protein composition even in non-fibrotic regions. Upon injury, regenerating epithelial cells deposit FN1, COLs, and matrix metalloproteinases (MMPs) into the basal lamina [60]. Of these, FN1 promotes ECM remodeling by providing a scaffold for COL deposition [61,62], further stabilized by cross-linking from transglutaminases and LOX. These cross-linking enzymes drive fibrillogenesis, increase stiffness, and promote EMT and senescence, creating a self-reinforcing niche that sustains the atypical ADI cell population [10,63]. Collectively, these changes yield spatially heterogeneous FF with distinct mechanical and biochemical cues, as shown in Figure 1.

Basal lamina remodeling is stage-dependent and dynamic, and early provisional matrix changes may be more plastic than later collagen-rich and highly cross-linked matrices [64,65]. In idiopathic pulmonary fibrosis, LOX family activity is linked to fibrillar collagen remodeling and matrix stiffening, which can reinforce transport barriers and sustain profibrotic epithelial states [66]. Early inflammatory and provisional remodeling phases occur during the first week, and more established fibrosis is commonly assessed around day 14 in bleomycin-injury models [67,68]. This timing suggests that nanoparticle-based interventions may have greater access and impact before extensive cross-linking and stiffening are established. Consistent with this concept, studies of fibrosis resolution support that matrix remodeling can regress under some conditions, whereas mature scar is generally harder to reverse [64]. Progressive COL deposition and LOX-mediated cross-linking can limit nanocarrier penetration through remodeled basal lamina [69,70,71].

#### 2.3.5. Matricellular Heterogeneity in the Fibroblastic Focus

Adding to this complexity is the cellular and interstitial ECM heterogeneity of FF. FF are organized interstitial structures containing macrophages, transformed “basaloid” epithelial cells, and collections of distinct fibroblast populations [10]. Within FF, multiple types of fibroblast populations have been identified that play major roles in interstitial fibrosis, alveolar invasion/destruction, and remodeling [72]. Moreover, the ECM deposition patterns are unique. The active edge of the FF actively produces FN1, tenascin C (TNC), serpin family H member 1, and versican (VCAN) [60]. With persistence of the FF, the acellular core is remodeled by deposition of glycoproteins, proteoglycans, COLs, and ECM regulators. COL III is the predominant COL isoform in areas of alveolar septal fibrosis, being replaced by COL I in regions of mature scar [69]. Importantly, LOX expression is upregulated; LOX is a copper-dependent enzyme that catalyzes the extracellular oxidative deamination of lysine COL residues, producing covalent cross-links [70]. Consistent with this, nascent FF are relatively compliant (Young’s modulus ~1.5 kPa), whereas mature scar can reach ~9.0 kPa [71]. Therefore, targeted strategies for drug delivery need to account for matricellular heterogeneity.

Additional bioactive ECM-associated components further shape fibrotic niche signaling and may also influence nanocarrier transport and binding within remodeled distal airways [73,74]. Osteopontin, encoded by SPP1, is enriched in profibrotic macrophage phenotypes in pulmonary fibrosis and is implicated in niche-level signaling that can reinforce epithelial and mesenchymal activation states [73]. Periostin is increased in idiopathic pulmonary fibrosis and is prominent in FF, where it supports myofibroblast-associated matrix remodeling activity [75]. Hyaluronan accumulation is also increased in idiopathic pulmonary fibrosis, and, as a highly hydrated glycosaminoglycan, it can contribute to a more gel-like local microenvironment that may affect diffusion and retention of inhaled nanocarriers [76].

Taken together, these factors indicate that effective therapies for IPF will involve delivering molecules that affect both dysplastic epithelium as well as the inflammatory alveolar fibroblast population. These constraints define functional requirements for inhalable systems; Section 3 outlines modalities and platforms to meet those requirements.

## 3. Nanoparticle-Based Strategies to Overcome Pulmonary Barriers

### 3.1. Therapeutic Modalities for Inhalation

**Figure 2:**
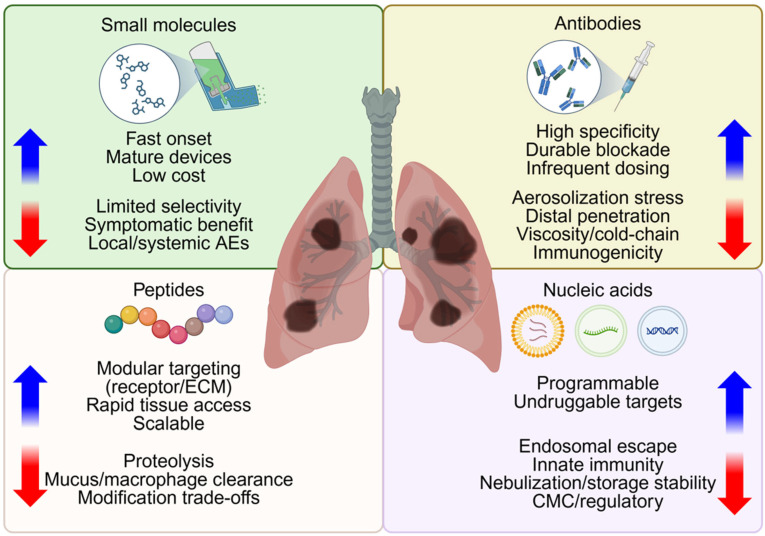
Considerations in selection of therapeutic modalities for inhalation therapy. Small molecules, antibodies, peptides, and nucleic acids are potential therapeutic modalities for nanoparticle delivery. This quadrant diagram highlights class-specific advantages (blue arrows) and potential limitations (red arrows) relevant to aerosolization, epithelial access, retention and clinical application of therapies for diseased lower airways. Created in BioRender. Lee, J. (2026) https://BioRender.com/t6efy0g (accessed on 22 January 2026).

In this section, therapeutic modality refers to the active entity, such as small molecules, antibodies, peptides, or nucleic acids, whether delivered as free drug or within a carrier. Building on a robust clinical experience in pulmonary therapeutics, this section reviews four inhaled therapeutic modalities, including small molecules, antibodies, peptides, and nucleic acids, and introduces the class contrasts summarized in Figure 2.

#### 3.1.1. Small Molecules

Small-molecule bronchodilators and corticosteroids are the most commonly used inhaled therapeutics in chronic obstructive lung disease because they provide rapid bronchodilation and anti-inflammatory effects through mature, low-cost inhaler devices [16]. These agents include short-acting (albuterol) and long-acting (salmeterol, formoterol) β2 agonists, long-acting muscarinic antagonists (tiotropium), and inhaled corticosteroids (budesonide, fluticasone). These drugs achieve fast onset and dose-proportional symptom relief, but are associated with local adverse events (dysphonia, oropharyngeal irritation, oral candidiasis) [77]. Since their primary functions are to relax airway smooth muscle or suppress airway inflammation, they provide symptomatic improvement rather than disease modification without directly targeting the fibrotic pathways that drive IPF [6,7].

In contrast, disease-modifying pharmacotherapy for IPF currently relies on the oral antifibrotics (pirfenidone and nintedanib) as mentioned above, which slow the forced vital capacity (FVC) decline and decrease acute exacerbation risk but do not reverse established fibrosis or cure the disease [6,12,13]. Since these agents must be dosed systemically to reach the distal fibrotic parenchyma, relatively high total doses are required, increasing off-target exposure and constraining combination strategies with additional small-molecule candidates [6,12]. These limitations have motivated efforts to develop inhaled small-molecule approaches that concentrate antifibrotic or anti-inflammatory drugs in the lower airways and alveolar regions while minimizing systemic burden [15,16,17,18]. However, many inhaled small molecules have short lung residence. Rapid dissolution and absorption can reduce distal-airway exposure, supporting retention-enhancing carrier or formulation designs [15,30].

Such emerging small molecules for IPF include epigenetic modulators, such as bromodomain and extra-terminal domain (BET) inhibitors that target bromodomain-containing protein 4 (BRD4) [78,79,80]. BRD4 integrates pro-inflammatory and pre-fibrotic transcriptional networks in lung fibroblasts and epithelial cells and is upregulated in experimental and human fibrotic lung tissue [80,81,82,83]. The pan-BET inhibitor JQ1, the clinical-stage BET inhibitor OTX015 (MK-8628), and newer BRD4 bromodomain 1 (BRD4-BD1)-selective compounds (ZL0516 and ZL0591) reduce myofibroblast transdifferentiation, ECM gene expression, and collagen deposition in vivo models of lung fibrosis [80,81,82,83,84]. Many BET/BRD4 inhibitors are highly hydrophobic and exhibit narrow therapeutic windows, necessitating enabling formulations to improve solubility, epithelial access, and lung retention [79,84]. These properties make BRD4 inhibitors attractive payloads for the inhalable nanocarrier platforms discussed in Section 3.2, which are designed to solubilize hydrophobic small molecules, enhance distal-airway deposition, and sustain local exposure at fibrotic niches [85,86,87,88,89].

#### 3.1.2. Antibodies

Antibodies with their pico-to-nanomolar affinity-based target specificity have transformed the management of severe eosinophilic and type-2-high asthma [90]. Representative examples include anti-IgE (omalizumab), anti-IL-5Rα (benralizumab), anti-IL-4Rα (dupilumab), anti-IL-5 (mepolizumab), and anti-TSLP (tezepelumab) [90]. However, the most-approved agents are given by systemic injections rather than by inhalation [91,92,93,94,95,96,97,98].

For IPF, antibody regimens have similarly focused on systemic inhibition of profibrotic pathways that are enriched in fibrotic lung tissue, including connective tissue growth factor (CTGF/CCN2), αvβ6-integrin-mediated activation of latent transforming growth factor-beta (TGF-β), and lysyl oxidase-like 2 (LOXL2)-mediated collagen cross-linking [69,70,71,99,100,101,102]. Pamrevlumab (FG-3019), a fully human monoclonal antibody against CTGF, slowed FVC decline and reduced fibrosis progression in phase 2 IPF studies but has not yet been approved [99,100]. BG00011, an anti-αvβ6 IgG1 antibody, suppressed TGF-β signaling biomarkers without improving FVC in a phase IIb trial [101]. Simtuzumab, a LOXL2-targeting antibody, also did not demonstrate clinical benefit in randomized phase 2 IPF trials [102]. Collectively, these systemic trials highlight that single-pathway antibody blockade has not yet produced a disease-modifying biologic for IPF but underscore the relevance of these targets and motivate lung-focused delivery strategies [99,100,101,102,103].

Inhaled and intranasal biologics, including antibodies and antibody fragments, are being explored to maximize epithelial and alveolar exposure while limiting systemic hurdles in respiratory diseases [17,19,90,103,104,105,106]. Formulation and delivery remain challenging because ~150 kDa proteins are sensitive to shear and air/liquid interfacial stress during nebulization, which can promote unfolding or aggregation [104,105]. High solution viscosity and limited tissue penetration in the distal lung further complicate deep-lung delivery [105]. These molecules offer highly specific, durable pathway blockade with infrequent dosing potential, yet their size and interfacial sensitivity complicate aerosolization and distal-epithelial penetration, add cold-chain and cost burdens, and necessitate immunogenicity monitoring [103,104,105,106].

Antibodies in idiopathic pulmonary fibrosis have reported limited efficacy or dose-limiting safety concerns in some studies, but this does not necessarily exclude antibody-based strategies. Inhaled delivery may increase local lung exposure while reducing systemic burden, although stability during aerosol generation remains critical because aggregation and activity loss can reduce delivered bioactivity [107,108,109].

#### 3.1.3. Peptides

Peptides are ~1–5 kDa in molecular weight and can diffuse to local epithelial or ECM targets more rapidly than larger biologics after deposition due to their small size. They can also be synthesized with precise sequence control to enable receptor- or ECM-targeted binding in disease-relevant cells [110,111,112]. In IPF, the fibrotic niche is enriched in collagen-rich, mechanically stiff ECM with altered epithelial signaling, which creates binding motifs that can be recognized by engineered peptides and peptide-decorated carriers [113,114,115,116].

For example, caveolin-1 scaffolding domain peptides demonstrate how inhaled peptides can be developed as disease-modifying IPF therapeutics [117,118]. The seven-amino acid peptide derived from the caveolin-1 scaffolding domain (CSP7) reduces apoptotic stress and profibrotic signaling in alveolar epithelial cells, attenuates established bleomycin-induced pulmonary fibrosis, and decreases ECM production in ex vivo human IPF lung tissue [117,118]. LTI-03, an inhaled caveolin-1-derived peptide clinical candidate, is being advanced as a dry-powder formulation for IPF, with emerging nonclinical and early clinical data supporting antifibrotic activity and acceptable tolerability when delivered directly to the distal lung [118,119]. A collagen-binding peptide (CBP8) labeled with ^68^Ga has been developed as a type I collagen-targeted positron emission tomography (PET) contrast agent [120,121]. It shows selective uptake in fibrotic lung regions in preclinical models and increased lung uptake in IPF patients, supporting peptide-based PET tracers as translational tools to quantify fibrotic matrix in vivo [113,114,115,116,120,121,122].

Despite the potential, unmodified peptides are susceptible to proteolysis and are removed by mucus trapping and macrophage clearance, which shortens residence time in the airways and alveolar regions [123,124,125]. Stabilization strategies such as cyclization, incorporation of D-amino acids, and hydrophilic shielding with polymers (poly(ethylene glycol) (PEG) or polysarcosine (pSar)) can improve protease resistance and reduce protein corona formation. However, they must preserve binding affinity, avoid excessive immunogenicity, and maintain aerosol performance [111,112,123,124,125]. Collectively, inhaled peptides offer IPF-relevant opportunities both as direct antifibrotic agents and as targeting ligands for nanocarriers and imaging probes, yet they require careful sequence and formulation engineering to balance stability, lung retention, and specific engagement of fibrotic niches [106,113,114,115,116,120,126].

#### 3.1.4. Nucleic Acids

Nucleic acids enable direct gene modulation, with small interfering RNA (siRNA) of ~21–23 bp and messenger RNA (mRNA) in the kilobase range [127,128]. Representative nanocarriers include ionizable lipid nanoparticles (LNPs) with helper lipids (distearoylphosphatidylcholine, cholesterol, and PEG lipids), as well as polymer carriers (poly(β-amino ester) polyplexes, Poly(amidoamine) (PAMAM) dendrimers, and poly(lactic-co-glycolic acid) (PLGA) nanoparticles). These protect nucleic acids from nucleases and promote endosomal escape for cytosolic delivery [19,127,128].

Nebulization can expose nucleic acid carriers to shear and air-to-liquid interfaces that may change particle properties and reduce cargo retention, which highlights the importance of device selection and formulation stabilization [129,130]. After deposition, fibrotic remodeling can further reduce effective delivery through transport limitations and increased macrophage clearance [131]. Endosomal escape remains a major efficiency bottleneck, so functional gene modulation should be confirmed after aerosolization in fibrosis-relevant models [131,132].

Nucleic acid therapies for pulmonary diseases are beginning to emerge. For example, an optimized inhaled ionizable LNP formulation that delivers mRNA encoding an interleukin-11-neutralizing antibody improves lung deposition, sustains local antibody expression, and attenuates experimental pulmonary fibrosis [19]. Another example is inhalable siRNA nanoparticles for Kirsten rat sarcoma viral oncogene (KRAS)-mutant non-small cell lung cancer. The aerosolized siRNA formulations achieve efficient lung deposition, robust KRAS knockdown, and tumor regression in rodent models, demonstrating that deep-lung delivery and functional gene silencing are feasible with clinically relevant aerosol devices [133,134]. In addition, thin-film freezing methods produce dispersible dry powders of plasmid DNA and other nucleic acids that retain activity after aerosolization and show high fine-particle fractions, enabling room-temperature-stable dosage forms for inhaled gene therapy [135,136].

These nucleic acid modalities are programmable and can be adapted to disease drivers that are difficult to target with small molecules or antibodies, including intracellular signaling nodes and transcriptional regulators enriched in fibrotic lung tissue [127]. Keys to success include efficient endosomal escape, control of innate immune activation, and preservation of activity through nebulization and storage, which together impose stringent requirements on chemistry, manufacturing, and controls [127,128].

### 3.2. Nanoparticle Platforms for Pulmonary Delivery

**Figure 3:**
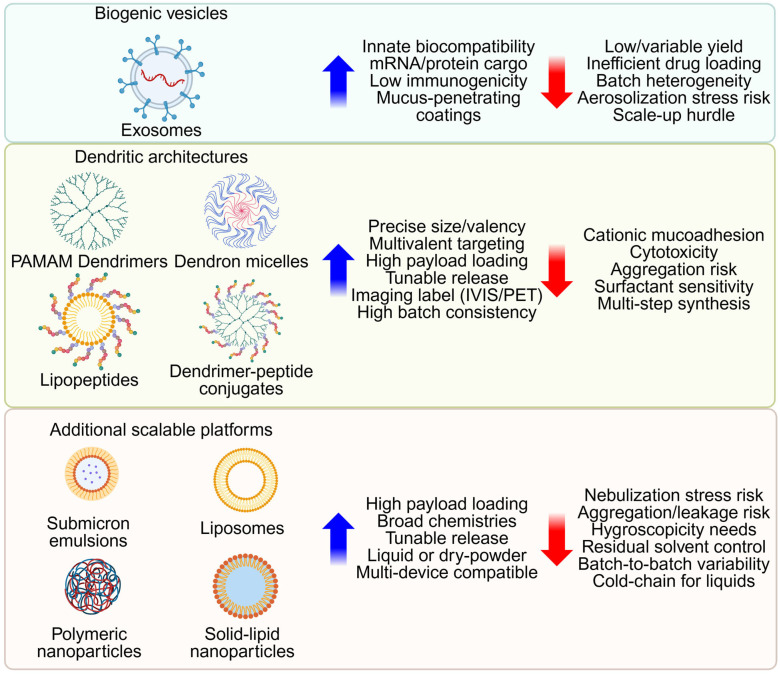
Platform benchmarking for inhaled nanomedicine. Biogenic vesicles, engineered dendritic architectures, and additional scalable platforms are organized under a common barrier-aware framework. For each family, key design parameters, biocompatibility, architectural precision, and manufacturing feasibility are compared. Created in BioRender. Lee, J. (2026) https://BioRender.com/smxxd73 (accessed on 22 January 2026).

We describe emerging nanoparticle platforms for pulmonary diseases, with an emphasis on novel nanocarriers under active development. Figure 3 benchmarks biogenic vesicles, engineered dendritic carriers, and other scalable systems within a common barrier-aware framework. Although these nanoscale carriers are below the MMAD range, matrix-binding or lesion-targeting surfaces, dry-powder forms (large porous particles or nano-in-microparticle matrices), and surface shielding with PEG, pSar, or other zwitterionic coatings can adapt them for deep-lung delivery and help overcome clearance and barrier limitations [137].

#### 3.2.1. Biological Nanovesicles Including Exosomes

Exosomes are nanoscale vesicles released by living cells with natural lipid-protein membranes that can carry mRNAs and proteins with inherent biocompatibility and immunomodulatory activity. Inhaled preparations have produced lung-repair and anti-fibrotic signals in preclinical models, and direct comparison studies have reported efficient delivery of protein and mRNA to bronchioles and lung tissue [138,139].

Despite these advantages, translation of exosomal drug delivery is limited by low and variable production yields, inefficient loading of exogenous drugs and nucleic acids, and batch-to-batch heterogeneity that complicates quality control and regulatory alignment [140,141]. Process innovations include parent-cell engineering to increase vesicle output or display targeting ligands and the development of exosome-synthetic nanoparticle hybrid systems that combine biological natural targeting with the scalability of polymer or lipid carriers [140,141].

To enable inhalation, recent studies pair exosome preparations with nebulizer-friendly buffers and device settings that preserve vesicle integrity and aerosol performance [139]. Formulations that reduce mucus adhesion, limit protein corona formation, and use low-fouling surface chemistry can improve epithelial access, reduce macrophage recognition, and extend residence in the lung [125,142,143]. Together, these strategies address mucus access, regional deposition in the lung, and retention. In parallel, engineered dendritic carriers provide complementary control over architecture, valency, and surface chemistry and are evaluated using the same barrier-aware criteria in Section 3.2.2 [144,145,146].

#### 3.2.2. Dendritic Architectures Including Dendron Micelles, Lipopeptides, and Dendrimer–Peptide Conjugates

Dendritic nanoparticles such as PAMAM dendrimers provide precise and uniform architectures and enable reproducible control over size, the number of ligands displayed on the surface, and internal cavities [144,145,146]. Dense functional groups support drug loading via conjugation and tunable release that can exceed many linear polymer carriers on a weight basis [144,145,147]. Particularly, multivalent display of peptides or antibodies increases avidity and can cluster receptors, which enhances apparent affinity and selectivity over free ligands, as demonstrated in our earlier studies [111,112,148]. This architectural precision enables reproducible ligand valency and sustained exposure, although highly cationic generations can increase mucin adhesion and membrane interaction unless neutralized during design [85,149,150].

Dendron-based amphiphiles and lipopeptide amphiphiles self-assemble into micelles with a dendritic or peptide shell and a hydrophobic core. These micelles can improve serum stability and provide more controlled release than linear micelles, and increase the solubility of hydrophobic payloads while allowing co-display of imaging or targeting motifs [86,87,147,151,152]. These micelles are typically small, tunable particles with improved serum stability and controlled release that favors distal-lung access, and there is clinical precedent for inhaled lipopeptide payloads, such as nebulized colistin, that achieve high local lung concentrations in resistant infections [153,154,155,156]. Since micelles disassemble below the critical micelle concentration and can be sensitive to shear and lung-fluid surfactants, formulations should secure colloidal integrity during aerosolization and after deposition [88,89].

Dendrimer–peptide conjugates (DPCs) extend this strategy by fixing targeting ligands and reporters on a single scaffold. DPCs that target MET achieved selective tumor imaging with high uptake and clear contrast by PET using zirconium-89 labels, and the design allowed control of the number of displayed ligands and pharmacokinetics [112,157]. The same platform strategy has blocked viral binding in independent studies where the inhibitory effect depended on dendrimer generation, showing how multivalency can translate to potent antagonism [148].

Together, these dendritic scaffolds support theranostic applications relevant to lung disease by coupling multivalent targeting, controlled pharmacokinetics, and quantitative imaging [112,148,158].

#### 3.2.3. Lipid and Polymer Nanoparticles

Given their maturity in pharmaceutical manufacturing and frequent use in inhaled drug development, lipid- and polymer-based nanoparticles are discussed with emphasis on aerosolization stability and deep-lung delivery constraints relevant to IPF.

Biodegradable or biocompatible materials include PLGA, PEG, poly(amine-co-ester), and polycaprolactone for slower release, together with natural polymers (alginate, gelatin, chitosan, albumin) [159]. They provide tunable release profiles and established manufacturing pathways for pulmonary medicines (asthma, anti-tuberculosis, anti-cancer) [159]. However, key constraints are their limited colloidal stability across aerosolization and manufacturability at scale [159,160,161,162,163]. In the IPF setting, inhalable polymeric or polymer-stabilized nanoparticles (pirfenidone, nintedanib, simvastatin, and other antifibrotic or pleiotropic agents) and dry-powder or nanosuspension formulations (nintedanib) have been advanced toward IPF therapies [16,23,164,165,166,167].

Bilayer vesicles typically containing various lipids and cholesterol are delivered as liquids or dry powders and can incorporate cell-penetrating peptides to increase cell uptake [168]. Inhaled liposomal formulations that achieve systemic effects have been reported for low-molecular-weight heparin, vasoactive intestinal polypeptide, insulin, parathyroid hormone, and calcitonin [169,170]. For fibrotic lung disease specifically, lipid nanocarriers co-encapsulating nintedanib with metformin or other metabolic modulators have reversed or attenuated bleomycin-induced pulmonary fibrosis in rodents [171,172,173,174,175].

Solid-lipid nanoparticles are built from solid lipids, surfactants, and water, and generally show lower cytotoxicity than many cationic polymer systems [176]. They enable multifunctionality, such as detection, diagnosis, imaging, ablation, and controlled release, with rapid in vivo degradation and have been aerosolized as nanosuspensions using jet or vibrating-mesh devices [177,178,179]. However, deep-lung performance depends on hygroscopicity, redispersion, and device compatibility, and clinical translation requires stable packages that preserve nanoscale integrity during aerosolization (see Section 3.2.6).

Overall, lipid and polymer nanoparticles provide a well-established foundation for inhaled antifibrotic delivery, but their successful translation in IPF ultimately depends on preserving nanoscale integrity and reproducible aerosol performance across devices.

#### 3.2.4. Additional Scalable Platforms

Other nanotherapeutic classes for inhaled delivery include submicron emulsions, liposomal nanocarriers, polymeric nanoparticles, and solid-lipid nanoparticles. Each of these is evaluated here under the same barrier-aware criteria for mucus access, aerodynamic placement, and retention, since these parameters determine whether IPF-relevant drugs can reach distal fibrotic regions rather than being lost in proximal airways [20,159].

Submicron emulsions have served as alternatives to liposomes for nucleic-acid and vaccine delivery to the airway (*Mycobacterium tuberculosis* DNA vaccines), with epithelial transfection driving antigen-specific T-cell stimulation [180]. Microemulsions (water-oil-surfactant systems) and nanoemulsions (kinetically stable dispersions of immiscible liquids) enable delivery of lipophilic, hydrophilic, and amphiphilic drugs, including first-line anti-tuberculosis agents (rifampicin, isoniazid, and pyrazinamide) [181,182,183,184,185]. These formulations offer high loading and scalable processing, although performance depends on stability under nebulization and in-lung fluids. For IPF, an inhaled deep eutectic solvent-based nanoemulsion of pirfenidone increased lung exposure, reduced markers of bleomycin-induced fibrosis, and allowed antifibrotic efficacy at reduced systemic doses [186,187].

Across these scalable carrier classes, relatively few inhaled nanoformulations have been evaluated in pulmonary fibrosis models, and even fewer have progressed toward clinical testing [20,188,189]. Beyond polymeric or lipid systems, preclinical fibrosis studies also include inorganic and hybrid nanoplatforms and metal-organic frameworks that enable high loading and engineered degradation [190,191,192]. However, expanding the platform amplifies the need to benchmark lung retention or clearance, biodegradation products, and immunotoxicity, especially in remodeled distal airways [192]. Collectively, these considerations support broader, barrier-aware platform selection and rigorous benchmarking for IPF-specific payloads and combination regimens [16,20,23].

#### 3.2.5. Aerosol Generation and Formulation Engineering

Building on the platform-specific considerations above, the clinical performance of inhalable nanoparticles depends strongly on how the aerosol is generated [15,17,22]. Nebulizers, dry-powder inhalers, and soft-mist inhalers can expose formulations to shear, air–liquid interfaces, and contact with device materials, which may promote aggregation, premature drug release, or cargo degradation [15,17,77,105,106]. These effects can change the emitted dose and the aerodynamic size distribution, particularly for nanosuspensions and formulations carrying biologics or nucleic acids [22,30,106]. Therefore, in-use stability and device compatibility testing should be integrated early in development. They should include practical assessments of factors such as adsorption to device components and concentration changes during nebulization [105,106,193]. Co-optimizing aerosol performance with nanoparticle integrity can improve dose reproducibility and support clinical translation [17,22,193].

Nano-in-micro designs embed nanoparticles inside respirable microparticles to improve dispersion and then recover nanoscale function after lung deposition [126,135,136]. Spray drying and thin-film freezing are scalable approaches to produce these powders and improve storage stability. Together, these aerosol and process constraints highlight why translation-oriented formulation choices matter beyond the nanocarrier itself and lead directly to the practical approaches summarized in Section 3.2.6 [17,22,194].

#### 3.2.6. Strategies to Improve Formulations for Clinical Translation

Rational design choices that reduce macrophage uptake and support sustained release can prolong residence time within pulmonary tissue, which would likely improve specificity and maximize localized therapeutic efficacy [39]. PEG, pSar, and zwitterionic coatings have been shown to reduce adhesive interactions with mucins and cell membranes [124,125,195,196,197].

Additionally, excipients stabilize fragile cargos during freezing, drying, and aerosolization. Glass-forming sugars and polyols preserve particle structure in the solid state and improve reconstitution [104,135]. Buffer strength, osmolality, and pH should minimize aggregation during nebulization, and gentle isotonic buffers preserve nucleic acids and proteins over the dosing interval [105].

As noted above, packaging of nanoparticles within larger porous matrices appears to be one of the most promising strategies, since it protects colloids during aerosolization and places the aerodynamic size in the about 1–5 µm window while maintaining the nanoscale features after deposition [135,198]. Nano-in-micro approaches based on spray drying or thin-film freezing achieve high fine-particle fractions and good redispersion to active nanospecies [135,136]. These powder and suspension forms then need devices and operating conditions that generate clinic-ready aerosols without loss of function [104,105,199].

Clinical translation of inhaled nanocarriers benefits from defining critical quality attributes that cover physicochemical stability and aerosol performance, including delivered dose uniformity and aerodynamic particle size distribution [200,201]. For nanocarrier systems, stability and impurity specifications should be supported by orthogonal characterization of size, morphology, drug loading, aggregation, and contaminants, together with stability-indicating assays [202,203]. Nano-in-micro dry-powder approaches add scale-up risk because spray drying and related processing can change nanoparticle integrity and lung aerodynamic performance, which motivates robust process understanding and scale-up strategies [194,204,205]. Device and formulation should be optimized and tested as a combined system since dose and particle size can be sensitive to device design and formulation properties [206,207]. For chronic inhalation, pulmonary tolerability and immune activation should be monitored. This is especially relevant for biological carriers such as exosomes and for cationic dendrimers where surface chemistry can influence inflammatory responses [139,208,209]. Representative inhaled nanomedicine candidates, patents, and clinical trials are summarized in Table 1, Table 2 and Table 3, respectively. At present, commercially available nanocarrier-based formulations are not available for idiopathic pulmonary fibrosis, and approved therapies remain orally administered small molecules.

## 4. Conclusions and Future Directions

Inhaled nanomedicine offers a practical route to treat lower airway disease by combining targeted deposition, navigation across mucus and cellular interfaces, and control of absorption and retention [135,136,142,143,198]. With understanding of the anatomical and biological barriers of fibrotic lungs, this review has summarized inhaled therapeutic modalities and nanocarrier platforms that have the potential for IPF treatments [138,139,140,141,144,145,146,147]. The objectives of the nanoformulations are to keep drug deposition in the gas-exchange region of the lung without damaging structure, navigate through pathologic mucus and remodeled matrix, and retain drug in disease regions with specificity [113,114,115,116,135,136,198].

Despite recent advances in inhaled delivery, disease-modifying treatments for IPF do not yet exist, and current oral antifibrotics only partially slow lung function decline [12,13,135,198]. As reviewed here, successful delivery to fibrotic gas exchange regions requires coordinated control of nanocarrier and payload parameters [30,41]. First, particle size and aerodynamic diameter should be tuned to favor deposition in small airways and alveoli, typically 1–5 μm [30,41]. Engineered nano-in-micro aerodynamic packaging that preserves nanostructure may place carriers in this window [135,198]. Second, surface chemistry should be engineered to cross viscous mucus and reduce nonspecific clearance by using mucus-penetrating or zwitterionic coatings [123,124,125,142,143,195]. In-use stabilization during nebulization together with macrophage evasion motifs would extend residence in the distal lung [39,193]. Third, targeting motifs for fibroblasts or matrix components should be incorporated to enrich deposition within IPF lesions [30,41]. Fourth, potent small molecules with nucleic acids or biologics could be combined to maximize local antifibrotic activity while limiting systemic exposure [15,16,127,128]. Embedding these principles within manufacturing, quality, and regulatory frameworks for inhaled and nanomaterial products is essential for translation to practical IPF treatments [193,200,212,213,214]. Future directions include patient stratification using imaging-defined phenotypes and adaptive trials that use PET endpoints for early go or no-go decisions. In parallel, scalable good manufacturing practice and nano-in-micro packaging for distal lung conditions will be essential to advance these inhaled nanomedicines into clinical use.

## Figures and Tables

**Table 1:** Representative inhaled nanomedicine candidates evaluated for IPF or pulmonary fibrosis-relevant models.

Candidate Class	Carrier and Cargo	Delivery	Stage	Model	Key Reported Outcome	Reference
Nucleic acid	Mucus penetrating lipid nanoparticles delivering dual mRNAs	Inhalation	Preclinical	Bleomycin induced pulmonary fibrosis (mouse)	Reduced fibrosis with functional recovery and epithelial restoration signals	[24]
Nucleic acid	Polymeric nanoparticles carrying siRNA targeting IL11	Inhalation	Preclinical	Bleomycin induced pulmonary fibrosis (mouse)	Antifibrotic effects with improved pulmonary function	[25]
Small molecule	ROS responsive liposomes carrying dimethyl fumarate	Inhalation	Preclinical	Fibrosis model (mouse)	Enhanced antifibrotic efficacy versus free drug and macrophage modulation	[173]
Small molecule	Liposomal nanoparticles co-loading verteporfin and pirfenidone (Lip@VP)	Atomized inhalation	Preclinical	IPF oriented models (mouse)	Improved lung function with reduced remodeling features	[210]
microRNA	Liposomes loaded with Hsa-miR-30a-3p	Inhalation	Preclinical	Bleomycin induced pulmonary fibrosis (mouse)	Attenuated fibrosis with functional improvement	[211]
Small molecule	Pulmonary surfactant based pirfenidone loaded nanovesicles	Inhalation	Preclinical	Bleomycin induced IPF model (mouse)	Reduced collagen and alpha SMA versus comparator formulations and oral pirfenidone	[175]

**Table 2:** Representative patent families of inhaled or pulmonary administered nanocarriers for pulmonary fibrosis, including idiopathic pulmonary fibrosis.

Patent	Platform and Payload	Relevance to Distal or Lower Airway Delivery in Pulmonary Fibrosis
WO2023036345A1	Aerosol-inhaled drug-loaded nanoparticles with siRNA payload	Describes inhaled nanoparticle delivery for pulmonary fibrosis with emphasis on mucus penetration and deep lung access
WO2023245176A1	Targeted nanoparticles, PDGFRB targeting, TXNDC5 inhibition using nucleic acid payloads	Describes targeted nanomedicine for fibrotic lung disorders with fibroblast directed delivery concepts relevant to fibrotic niche targeting
WO2024041415A1	Collagenase liposome inhalant	Describes liposomal collagenase formulations intended for inhalation use in pulmonary fibrosis
WO2019209787A1	Inhalable liposomal sustained release composition, includes tyrosine kinase inhibitor examples	Describes aerosolized liposomal compositions for idiopathic pulmonary fibrosis treatment, supporting inhaled liposomal antifibrotic approaches
WO2022074181A1	Inhaled nanocarrier formulations, including lipid nanoparticles	Describes inhalation use of lipid nanoparticles in lung disorders including idiopathic pulmonary fibrosis, with examples in fibrosis models

**Table 3:** Representative ongoing and completed inhaled clinical trials relevant to idiopathic pulmonary fibrosis.

ClinicalTrials.gov ID	Intervention	Phase	Status	Primary Focus or Endpoint
NCT04708782	Inhaled treprostinil (TETON-1)	3	Active, not recruiting	Change in absolute FVC at week 52
NCT06329401	Pirfenidone solution for inhalation (AP01)	2b	Recruiting	Safety and efficacy over 52 weeks with lung function endpoints
NCT03832946	Inhaled GB0139 (galectin-3 inhibitor)	2b	Completed	Efficacy and safety evaluation over 52 weeks

## Data Availability

Not applicable.

## Abbreviations

The following abbreviations are used in this manuscript:

IPF	Idiopathic pulmonary fibrosis
COPD	Chronic obstructive pulmonary disease
AA	Allergic asthma
M	Million
ECM	Extracellular matrix
EMT	Epithelial–mesenchymal transition
ADI	Alveolar differentiation intermediate
FN	Fibronectin
LOX	Lysyl oxidase
MUC	Mucin
MMAD	Mass median aerodynamic diameter
LAM	Laminin
COL	Collagen
TNC	Tenascin C
VCAN	Versican
FVC	Forced vital capacity
BET	Bromodomain and extra-terminal domain
BRD	Bromodomain-containing protein
BD	Bromodomain
CTGF	Connective tissue growth factor
TGF-β	Transforming growth factor-beta
LOXL	Lysyl oxidase-like
PET	Positron emission tomography
PEG	Poly(ethylene glycol)
pSar	Polysarcosine
siRNA	Small interfering RNA
mRNA	Messenger RNA
LNP	Lipid nanoparticle
PAMAM	Poly(amidoamine)
PLGA	Poly(lactic-co-glycolic acid)
KRAS	Kristen rat sarcoma viral oncogene
DPC	Dendrimer–peptide conjugate
FF	Fibroblastic foci
MMP	Metalloproteinases
